# Low CDX2 expression and its clinicopathological associations in colorectal adenocarcinoma: prognostic insights from a retrospective cross-sectional study in Vietnam

**DOI:** 10.1186/s13000-025-01734-y

**Published:** 2025-12-12

**Authors:** Huy Minh Le, Tram Ho Ngoc Le, Hanh Thi Tuyet Ngo, Thao Thi Thu Luu, Thang Quoc Pham, Giang Huong Tran, Thien Thanh Ly

**Affiliations:** 1 Department of Histology, Embryology, and Pathology, University of Medicine and Pharmacy at Ho Chi Minh City, 217 Hong Bang Street, Cho Lon Ward, Ho Chi Minh City, 700000 Vietnam; 2 Department of Pathology, University of Medicine Center Ho Chi Minh, 215 Hong Bang Street, Cho Lon Ward, Ho Ho Chi Minh City, 700000 Vietnam

**Keywords:** CDX2, Colorectal adenocarcinoma, Clinicopathological features, Prognosis

## Abstract

**Background:**

CDX2, an intestine-specific transcription factor, is essential for colorectal epithelial differentiation and has been widely studied as a biomarker in colorectal adenocarcinoma (CRC). However, most previous studies applied a binary evaluation (positive/negative), which may underestimate its clinical significance.

**Methods:**

We conducted a retrospective cross-sectional study of 356 surgically resected CRC cases at the University Medical Center, Ho Chi Minh City. CDX2 expression was evaluated by immunohistochemistry using an immunoreactivity score (IRS) that combined staining ratio and intensity. Associations between CDX2 expression and clinicopathological features were analyzed using chi-square and logistic regression tests.

**Results:**

High CDX2 expression was observed in 88.8% of tumors, whereas 11.2% showed low expression. Low CDX2 was significantly associated with poor histological differentiation (OR = 3.79; 95% CI: 1.11–12.93; *p* = 0.033) and advanced local stage pT4a–pT4b compared with pT1–pT3 (OR = 2.86; 95% CI: 1.47–5.58; *p* = 0.002). No significant associations were found with patient age or sex. The combined scoring system allowed clearer discrimination between biologically distinct subgroups than the traditional binary method.

**Conclusions:**

Low CDX2 expression is linked to aggressive pathological features and advanced tumor stage in CRC, highlighting its clinicopathological associations. Semi-quantitative evaluation of CDX2 using both staining ratio and intensity provides a more informative assessment that may aid risk stratification and guide clinical decision-making in CRC patients.

## Highlights

• A large cross-sectional analysis of 356 Vietnamese colorectal adenocarcinomas.

• CDX2 assessed by combined staining ratio and intensity rather than binary classification.

• Low CDX2 significantly associated with poor differentiation and pT4 stage.

• The combined scoring method improves clinicopathological stratification.

• Findings support the clinicopathological relevance and potential clinical utility of CDX2.

## Introduction

With roughly 1.93 million new cases and 935,000 deaths in 2020, colorectal cancer (CRC) is one of the most frequent and deadly cancers globally. By 2040, it is projected to increase by 63% in incidence and 73% in mortality [1, 2]. CRC was a significant contributor to more than 120,000 cancer-related fatalities that occurred in Vietnam in 2022 [3]. Caudal-type homeobox 2 (CDX2) is a key transcription factor for intestinal differentiation and is routinely applied as a sensitive and specific immunohistochemical marker for gastrointestinal epithelial origin [4]. Recent studies suggest CDX2 expression may also serve as a clinicopathological associations in colorectal adenocarcinoma [5, 6].

Several studies have shown significant correlation between CDX2 expression and biological behavior of CRC. Dalerba et al., in a study of more than 2,000 CRC cases, found that reduced or absent CDX2 expression was strongly associated with advanced disease and poor prognosis [7]. Jahil et al., in a series of 63 Iraqi CRC patients, found that low CDX2 expression was associated with shorter mean overall survival (17.9 vs. 33.4 months) and correlated with adverse features like older age, mucinous histology, poor differentiation, deeper invasion, nodal involvement and distant metastasis [8]. Singh et al. [9] and Xu et al. have also confirmed the prognostic role of CDX2 loss in Indian and Chinese cohorts respectively [10]. Given these findings, our study focused on combining tumor stage with CDX2 expression in surgically treated Vietnamese CRC patients. The goal was to provide solid data to refine patient grouping and to better predict prognosis so that we can have more personalized treatment and better survival outcome.

Previous studies have used a binary classification (positive vs. negative) to assess CDX2 expression [7, 11]. This approach being particularly reported in studies from Vietnam. Notably, a study by Chu Van Duc et al. documented such findings in the form of a thesis, although it has not been published in a peer-reviewed journal. However, this approach does not fully reflect the actual protein expression [7]. Tumors with weak or focal CDX2 staining are still considered “positive” although low CDX2 expression is associated with unfavorable prognosis and poor survival [7, 8, 10, 12]. This may lead to misinterpretation of its true prognostic value in clinical practice. We need a more refined method to assess CDX2 expression that integrates both the proportion of tumor nuclei showing staining and the staining intensity [1, 8–10, 13]. Therefore, we did this study to classify colorectal adenocarcinomas into high and low expression groups using a semi-quantitative scoring system and to evaluate the association of CDX2 expression with histological differentiation and tumor progression.

## Materials and methods

### Study design and patients

This was a retrospective, cross-sectional descriptive study conducted at the Department of Pathology, University of Medicine Center Ho Chi Minh, Ho Chi Minh City, in collaboration with the Department of Histology, Embryology and Pathology, University of Medicine and Pharmacy at Ho Chi Minh City. The study period was from July 2024 to July 2025.

A total of 356 consecutive patients with surgical resection for histologically confirmed colorectal adenocarcinoma in 2023 were included. Inclusion criteria were:

- Primary colorectal adenocarcinoma.
- Availability of formalin-fixed paraffin-embedded (FFPE) tissue blocks with sufficient tumor tissue for additional hematoxylin–eosin (H&E) and immunohistochemical staining.
- Complete clinicopathological data including age, sex, tumor site, histological subtype, differentiation, pT and pN stage, and distant metastasis status.

Exclusion criteria include patients who received preoperative chemotherapy or radiotherapy, had multiple primary malignancies, or whose tissue blocks were poorly preserved.

### Tissue microarray construction

Representative tumor areas were identified on H&E–stained slides by two pathologists. Tissue microarrays (TMAs) were constructed using 4.0 mm cores, with at least two representative tumor cores per case to minimize sampling bias [14]. TMA is a reliable and widely accepted tool for quantitative and comparative analyses in modern pathological research [15]. Although no quantitative comparison between the two cores was performed, concordance was confirmed during evaluation, and in cases of minor variation, the core with the more representative staining was used [16].

Immunohistochemistry.

Four-micron-thick TMA sections were cut and mounted on charged slides. After deparaffinization and rehydration, antigen retrieval was performed using citrate buffer (pH 6.0) in a pressure cooker. Immunohistochemistry for CDX2 was performed using a rabbit monoclonal antibody against CDX2 (clone EPR2764Y, ready-to-use, Ventana/Roche, cat. 760–4380). Detection was carried out on an automated immunostainer (Ventana BenchMark ULTRA, Roche Diagnostics) using an OptiView DAB detection kit. Normal colonic mucosa was used as the positive control. Only nuclear staining was considered specific/positive.

### Evaluation of CDX2 expression

Evaluation of CDX2 immunostaining was performed independently by two experienced gastrointestinal pathologists, each blinded to clinical and outcome data. A pathology resident participated as a recorder and trainee, entering data and observing the evaluation process. In cases of discrepancy between the two senior pathologists, slides were jointly re–examined at a multi-headed microscope until consensus was achieved. This consensus-based approach reflects current best practice in immunohistochemistry and ensured methodological rigor while also providing an educational opportunity. Inclusion of a third pathologist for inter-observer agreement (κ) analysis is planned for future validation.

Expression was scored semi-quantitatively by combining two parameters:

- Staining ratio: proportion of positive tumor cell nuclei (0 = 0%, 1 = 1–25%, 2 = 26–50%, 3 = 51–75%, 4 = > 75%).
- Staining intensity: compared to normal colonic epithelium (0 = negative, 1 = weak, 2 = moderate, 3 = strong).

We applied the immunoreactivity score (IRS) system by multiplying the staining ratio (0–4) and intensity (0–3), yielding a total score of 0–12. For dichotomization, we adopted a cut–off of 5 points to define high (≥ 5) versus low (≤ 4) CDX2 expression.

This threshold has been employed in previous studies of colorectal carcinoma [9, 10] and other transcription factor markers [7, 12]. Although any dichotomization may be somewhat arbitrary, the threshold of 5 balanced staining proportion and intensity and was consistent with the distribution of our series, thereby providing clinically meaningful stratification.

### Clinicopathological assessment

Histological classification and grading followed the WHO Classification of Digestive System Tumours, 5th edition (2019) [5]. Pathological staging was based on the AJCC/UICC TNM 8th edition [17]. Other histological parameters recorded included tumor budding, tumor-infiltrating lymphocytes (TILs), lymphovascular invasion, perineural invasion, necrosis, and presence of satellite nodules [18].

### Statistical analysis

Data were collected using Epidata and analyzed with SPSS version 20.0 (IBM Corp., Armonk, NY, USA). Associations between CDX2 expression and categorical variables were examined using chi-square or Fisher’s exact test, as appropriate. Odds ratios (OR) with 95% confidence intervals (CI) were calculated. Logistic regression was performed to adjust for potential confounders. A p-value < 0.05 was considered statistically significant.

### Ethical approval

The study was approved by the Ethics Committee of the University of Medicine and Pharmacy at Ho Chi Minh City (Approval No. 118/2024/HĐ-ĐHYD). Written informed consent was obtained from all patients at the time of surgery for use of their archived tissue samples in research.

## Results

### Patient characteristics

**Table 1:** Clinicopathological characteristics of 356 patients with colorectal adenocarcinoma

Variable	Category	*n*	%
Age	< 40 years	22	6.2
	≥ 40 years	334	93.8
Sex	Male	188	52.8
	Female	168	47.2
Tumor location	Right colon	75	21.1
	Left colon	182	51.1
	Rectum	99	27.8
Gross morphology	Ulceroinfiltrative	137	38.5
	Exophytic	122	34.3
	Others (polypoid, annular, etc.)	97	27.2
Histological type	Adenocarcinoma NOS	302	84.8
	Mucinous carcinoma	35	9.8
	Serrated adenocarcinoma	14	3.9
	Signet ring cell carcinoma	3	0.9
	Adenoma-like carcinoma	2	0.6
Grade	Well-differentiated	13	3.7
	Moderately differentiated	330	92.6
	Poorly differentiated	13	3.7
pT stage	T1–T3	247	69.4
	T4a–T4b	109	30.6
Nodal status	N0	175	49.2
	N1	126	35.4
	N2	55	15.4
CDX2 expression	High (IRS ≥ 5)	316	88.8
	Low (IRS ≤ 4)	40	11.2

As shown in Table 1, among 356 patients, 93.8% were ≥ 40 years, while only 6.2% were younger than 40. Males accounted for 52.8% of the cohort, resulting in a male-to-female ratio of approximately 1.1:1.

Tumor location was most frequently in the left colon (51.1%), followed by rectum (27.8%) and right colon (21.1%). Macroscopically, ulceroinfiltrative (38.5%) and exophytic (34.3%) growth patterns predominated, while polypoid and annular morphologies were less common.

Histologically, adenocarcinoma not otherwise specified (NOS) was the most common type (84.8%). Mucinous carcinoma accounted for 9.8%, serrated adenocarcinoma for 3.9%, signet ring cell carcinoma for 0.9%, and adenoma-like carcinoma for 0.6%. Regarding grade, moderately differentiated tumors were most common, while poorly differentiated carcinomas accounted for 3.7%.

### CDX2 expression

**Fig. 1:**
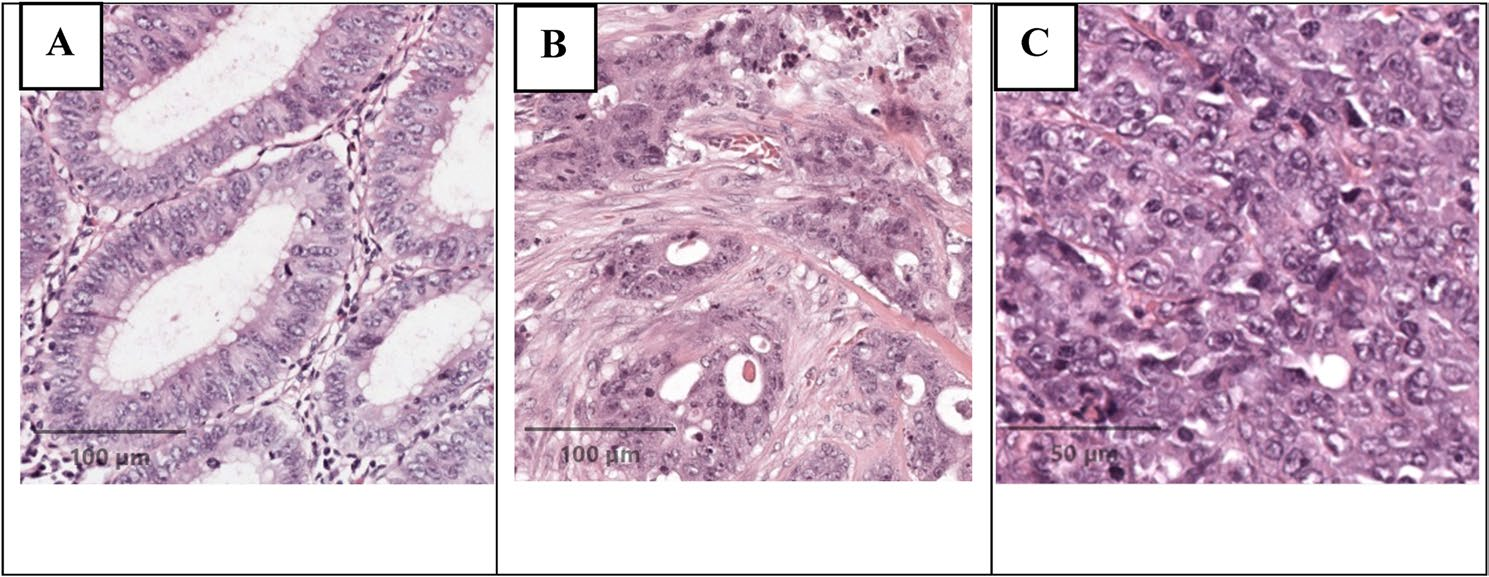
Representative histology of colorectal adenocarcinoma. (**a**) Well-differentiated adenocarcinoma with glandular formation (H&E, ×100). (**b**) Moderately differentiated adenocarcinoma with irregular, fused glands and intermediate nuclear atypia (H&E, ×200). (**c**) Poorly differentiated adenocarcinoma showing solid growth pattern and marked nuclear atypia (H&E, ×200)

CDX2 expression was high in 316 cases (88.8%) and low in 40 cases (11.2%). Representative H&E and CDX2 IHC staining patterns are illustrated in Fig. 1.

**Fig. 2:**
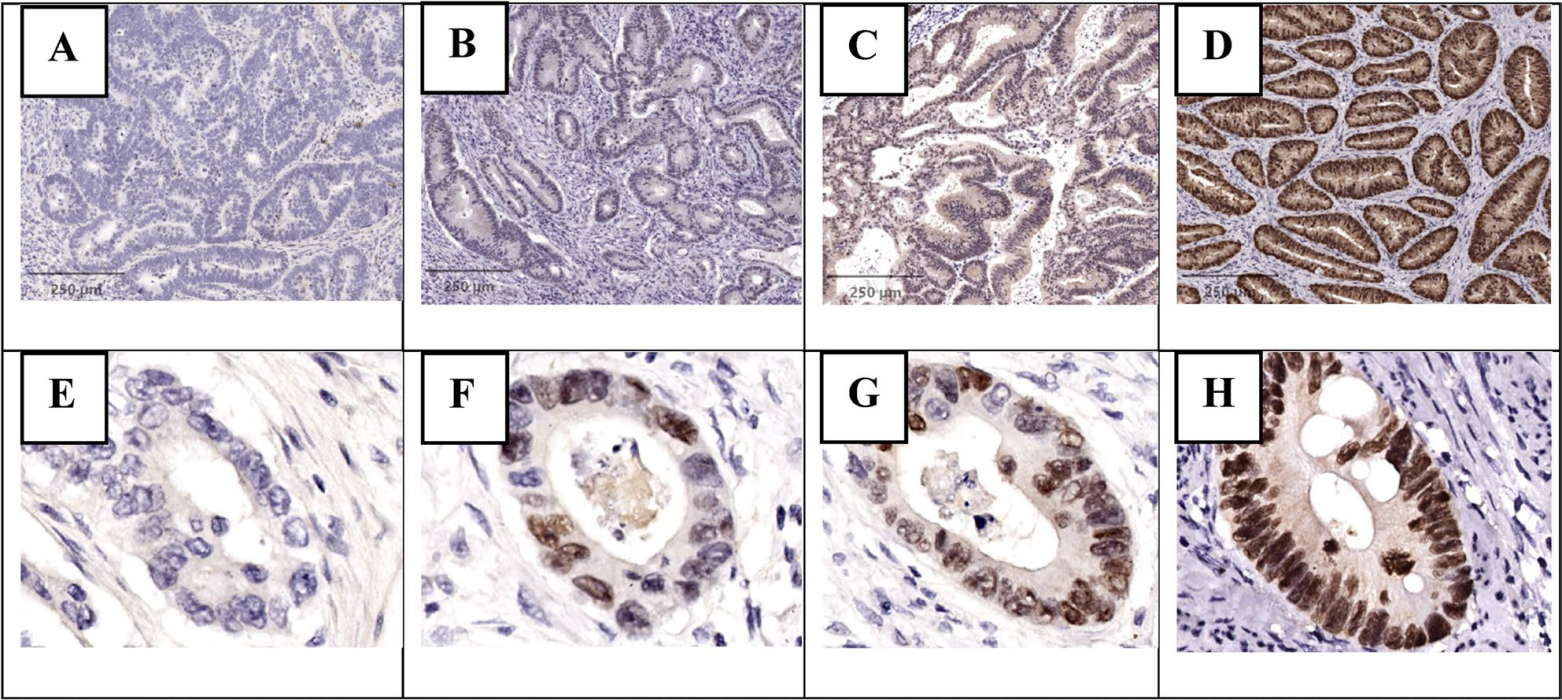
Immunohistochemical staining for CDX2. **A**-**D** Staining ratio: proportion of positive tumor cell nuclei (**A**. 0%, **B**. 1–25%, **C**. 26–50%, **D**. 51–100%) (IHC, ×40). **E**-**H** Staining intensity: compared to normal colonic epithelium (**E**. negative, **F**. weak, **G**. moderate, **H**. strong) (IHC, ×200). This semi-quantitative approach was used to generate the IRS scoring system (0–12) applied in this study. All images were captured at consistent magnifications, and intensity grading reflects true chromogenic strength rather than differences in tumor cell number

### Associations with clinicopathological parameters

**Table 2:** Association between CDX2 expression and clinicopathological features

Variable	CDX2-high (%)	CDX2-low (%)	OR (95% CI)	*p*-value
Age	< 40: 86.4	13.6	1.27 (0.36–4.49)	0.71
Sex	Female: 85.7	14.3	1.79 (0.91–3.50)	0.09
Tumor location	Left colon/others: 95	5	0.76 (0.39–1.47)	0.412
Gross morphology	Ulceroinfiltrative/others: 92.7	7.3	0.50 (0.23–1.05)	0.067
Histological type	Mucinous/others: 85.7	14.3	1.36 (0.50–3.74)	0.549
Differentiation	Poor/others: 69.2	30.8	3.79 (1.11–12.93)	0.033
pT stage	T4/others: 80.6	19.4	2.86 (1.47–5.58)	0.002
Nodal status	N2/others: 89.1	10.9	0.96 (0.38–2.41)	0.93

Low CDX2 expression was significantly associated with poor differentiation (*p* = 0.033). Similarly, low CDX2 was more frequent in advanced tumor stage pT4 compared to pT1–T3 (*p* = 0.002). Logistic regression analysis confirmed that CDX2–low status increased the odds of poor differentiation by approximately 3.8–fold and advanced T stage by 2.9–fold (Table 2). No significant associations were observed between CDX2 expression and age, sex, gross morphology, histological type or nodal status.

## Discussion

This retrospective cross-sectional study included 356 Vietnamese patients. Our findings show that poor differentiation and advanced local tumor stage are highly associated with reduced CDX2 expression, despite the fact that it is rather uncommon (11.2%). The clinicopathological characteristics of our cohort are largely consistent with previous studies, supporting the generalizability of our results. Our clinicopathological characteristics were largely comparable with previous studies from Asia and Europe [1, 8–10], supporting the representativeness of our cohort. Notably, we discovered a strong correlation between poor differentiation and low CDX2, supporting earlier findings from China, India, and Iraq [8–10].

1. CDX2 loss and aggressive features
  Our positivity rate of 88.8% exceeds those from Iraq (52.4%) [8], China (47.8%) [10], India (66.4%) [9]. This difference is likely due to methodological factors: while most earlier studies used binary scoring system (positive vs. negative), we applied a semi-quantitative immunoreactivity score (IRS) combining staining ratio with intensity. Previous authors have emphasized that binary evaluation may mislabel weak CDX2 expression as positive, potentially underestimating prognostic impact [7, 12]. By refining classification, our study highlights a distinct CDX2-low subgroup with adverse histological features. CDX2 is a transcription factor critical for intestinal differentiation. Its downregulation may trigger epithelial-mesenchymal transition (EMT), dedifferentiation, and invasive growth [5, 12]. CDX2 loss often overlaps with microsatellite instability (MSI) and CpG island methylator phenotype (CIMP), particularly in right-sided tumors [5]. Our findings that CDX2-low tumors cluster with poor differentiation and higher T stage support its role as a marker of tumor aggressiveness.
2. Methodological strength: semi-quantitative scoring
  One methodological strength of our study is the use of a semi-quantitative IRS that incorporates both the proportion of positive tumor nuclei and staining intensity, rather than the commonly employed binary positive/negative approach. We selected an IRS cut-off of 5 to classify CDX2 expression into high and low categories. This threshold has been applied in previous studies of colorectal carcinoma, including those by Singh et al. and Xu et al. [9, 10], and has also been used in other tumor types for transcription factor evaluation [7, 12]. Although any dichotomization may be considered arbitrary, this threshold provided a pragmatic balance and effectively stratified our series into clinically meaningful groups, with CDX2-low tumors showing strong associations with poor differentiation and advanced stage. Another methodological strength is that CDX2 scoring was performed independently by two pathologists, with discrepant cases resolved by consensus review, thereby minimizing interobserver variability. While alternative approaches such as median-based or ROC-derived thresholds could be applied, we selected an IRS cut-off of 5 for consistency with prior publications [9, 10] and because it reflected the distribution of our study population. Our TMA-based evaluation, though subject to potential sampling bias, showed high concordance between cores and followed validated protocols [14, 19]. CDX2 immunohistochemistry is inexpensive, widely available, and can stratify colorectal cancer patients into prognostic subgroups [20]. Dalerba et al. [7] demonstrated that CDX2-negative patients may derive greater benefit from adjuvant chemotherapy.
3. Limitations and future perspectives
  There are several limitations to this study. First, its retrospective cross-sectional design does not allow survival analysis or causal inference. Although the large sample size and use of tissue microarrays increased reliability, selection bias may occur because only surgically resected patients without prior neoadjuvant therapy were included. In addition, molecular markers such as MSI or CIMP status were not evaluated, which limited our ability to investigate the underlying mechanism of CDX2 loss [21]. Another limitation is the use of tissue microarrays, which may not fully capture intratumoral heterogeneity of CDX2 expression; however, two representative cores per case were included to minimize sampling bias [19]. Some odds ratios in our logistic regression analyses exhibited wide confidence intervals, reflecting limited event numbers in certain subgroups; these findings should therefore be interpreted with caution. Finally, while interobserver variability was minimized through independent double-review and consensus, our results should be confirmed in larger, multicenter cohorts with survival follow-up and integrated molecular correlation.

Future research should focus on validating our semi-quantitative scoring system in independent cohorts, ideally with survival follow-up to confirm the prognostic utility of CDX2. Integration of CDX2 with molecular markers such as MSI [22], p53 [16], or E-cadherin [23] may further refine CRC classification and prognostic stratification [24]. Prospective, multicenter studies in Vietnam and other Asian countries would help determine whether regional differences in CDX2 expression patterns exist and how they impact treatment decisions. Moreover, translational research is necessary to explore whether CDX2 status can be a predictive factor for response to chemotherapy or immunotherapy, potentially guiding personalized therapy [7, 25].

All immunostaining used the automated Ventana platform to ensure procedural consistency. However, future cross-platform validation (e.g., Leica Bond) is warranted to confirm reproducibility. Future multicenter studies with long-term follow-up on survival and recurrence are needed to confirm the independent prognostic value of CDX2 across more diverse patient populations. Survival analyses from international cohorts have consistently demonstrated that patients with low CDX2 expression have a 2- to 5-fold higher risk of mortality and a significantly shorter overall survival compared with those who have high CDX2 expression [9, 10, 26–28]. Therefore, incorporating longitudinal outcome data in future cohorts will be essential to clarify the independent prognostic role of CDX2 in colorectal cancer. Receiver operating characteristic (ROC) curve analysis could be applied to determine the optimal IRS threshold for distinguishing “high” versus “low” CDX2 expression, thereby enhancing clinical applicability. In addition, AI-assisted quantitative analysis of immunohistochemical staining may help reduce subjective bias and improve inter-laboratory reproducibility [28]. Finally, the integration of CDX2 with molecular biomarkers such as MSI, RAS, BRAF, or SATB2 [27] may lead to the development of comprehensive prognostic and predictive models, supporting a more personalized therapeutic approach in colorectal cancer.

In summary, our study demonstrates that low CDX2 expression, evaluated by a combined scoring system of staining ratio and intensity, is significantly associated with poor differentiation and advanced local stage in Vietnamese CRC patients. These findings support the biological role of CDX2 in maintaining intestinal differentiation and its clinicopathological associations. While survival validation is needed, our work provides a methodological improvement and a foundation for incorporating CDX2 scoring into routine pathology practice and risk stratification models in CRC.

## Conclusion

Low CDX2 expression was significantly correlated with poor differentiation and advanced stage. Compared with the binary method, semi-quantitative scoring offered clearer stratification, underscoring its potential as a practical biomarker for prognostic classification in colorectal adenocarcinoma.

## Data Availability

The datasets generated and/or analyzed during the current study are not publicly available due to patient confidentiality but are available from the corresponding author on reasonable request.
